# Tan's Epsilon-Determinant and Ranks of Matrices over Semirings

**DOI:** 10.1155/2015/242515

**Published:** 2015-02-04

**Authors:** Preeti Mohindru, Rajesh Pereira

**Affiliations:** Department of Mathematics & Statistics, University of Guelph, Guelph, ON, Canada N1G 2W1

## Abstract

We use the *ϵ*-determinant introduced by Ya-Jia Tan to define a family of ranks of matrices over certain semirings. We show that these ranks generalize some known rank functions over semirings such as the determinantal rank. We also show that this family of ranks satisfies the rank-sum and Sylvester inequalities. We classify all bijective linear maps which preserve these ranks.

## 1. Introduction

There are many equivalent ways of defining the rank of a matrix over a field. The rank of an *m* by *n* matrix *A* could be defined as the largest *k* for which there exists a *k* by *k* submatrix of *A* with nonzero determinant, or the dimension of the row space of *A*, or the dimension of the column space of *A* or the smallest *k* for which there exists an *m* by *k* matrix *B* and a *k* by *n* matrix *C* with *A* = *BC*. For matrices over semirings, all of these definitions are no longer equivalent and each of these generalizes to a distinct rank function for matrices over semirings. There are many different rank functions for matrices over semirings and their properties and the relationships between them have been much studied (see, e.g., [1–3]). In this paper, we use the *ϵ*-determinant of Tan [4, 5] to define a new family of rank functions for matrices over semirings. We examine the properties of these rank functions as well as their relationship to some of the other rank functions found in the literature.

In this section, we review some background material on semirings. In Section 2, we list the definition and some important properties of *ϵ*-determinant of Tan first introduced in [4, 5]. We then use the *ϵ*-determinant to introduce a new family of rank functions called the (*ϵ*, *I*)-rank functions and show that these rank functions satisfy some of the usual inequalities such as the rank-sum inequality and the Sylvester inequality. In Section 3, we look at bijective linear preservers of (*ϵ*, *I*)-rank for all matrices over commutative antinegative semirings. In Section 4, we introduce the sign pattern semiring and show that our rank function in this case is equal to the dimension of the largest sign-nonsingular submatrix. Our new rank functions depend on the ideal structure of the semirings and this leads us to study semirings which have a unique maximal ideal in Section 5. We show how the (*ϵ*, *I*)-rank generalizes determinantal rank in Section 6.

Semirings are a generalization of rings. Semirings satisfy all properties of unital rings except the existence of additive inverses. Vandiver introduced the concept of semiring in [6], in connection with the axiomatization of the arithmetic of the natural numbers.

**Definition 1 (see [7]).**

A semiring is a set *S* together with two operations ⊕ and ⊗ and two distinguished elements 0, 1 in *S* with 0 ≠ 1, such that

(1) (*S*, ⊕, 0) is a commutative monoid,
(2) (*S*, ⊗, 1) is a monoid,
(3) ⊗ is both left and right distributive over ⊕,
(4) the additive identity** 0** satisfies the property *r* ⊗ 0 = 0 ⊗ *r* = 0, for all *r* ∈ *S*.

In other words, semirings are unital rings without the requirement that each element has additive inverse. If (*S*, ⊗, 1) is a commutative monoid then *S* is called a* commutative semiring*. A semiring is said to be* antinegative* or* zerosumfree* if the only element with an additive inverse is the additive identity 0. An element of a semiring *S* is called a* unit* if it has a multiplicative inverse in *S*.

The natural numbers form a semiring under the usual addition and multiplication. A much studied example of a semiring is* the max-plus semiring *(*ℝ*_max⁡_, ⊕, ⊗), where *ℝ*_max⁡_ = *ℝ* ⋃ {−*∞*} with *a* ⊕ *b* = max⁡{*a*, *b*} and *a* ⊗ *b* = *a* + *b*. In this case 0 = −*∞* and 1 = 0 [8]. A totally ordered set *S* with greatest element** 1** and least element** 0** forms a semiring with *a* ⊕ *b* = max⁡{*a*, *b*} and *a* ⊗ *b* = min⁡{*a*, *b*}. This is called* chain semiring*. The chain semiring with two elements {0, 1} is called the* Boolean semiring* and it is denoted by *β*.

Let *M*_*m*,*n*_(*S*) denote the set of all *m* by *n* matrices over *S* and *M*_*n*_(*S*) denotes the set of all *n* by *n* matrices over *S*. Addition and multiplication of these matrices can be defined in the usual way. Let *A* be an *m* by *n* matrix. Then the element *a*_*ij*_ is called the (*i*, *j*)-entry of *A*. The (*i*, *j*)-entry of *A* is sometimes denoted by *A*_*ij*_. Let *A* = [*a*_*ij*_] and *B* = [*b*_*ij*_] be *m* by *n* matrices over a semiring (*S*, ⊕, ⊗) and let *C* = [*c*_*ij*_] be an *n* by *p* matrix over the same semiring. Then *A* + *B* = [*a*_*ij*_ ⊕ *b*_*ij*_] and *AC* = [⨁_*k*=1_^*n*^ *a*_*ik*_ ⊗ *c*_*kj*_]. The set of *n* by *n* matrices over a semiring is itself a semiring. For any *k* ∈ *S*, the matrix *kA* = [*k* ⊗ *a*_*ij*_].

**Definition 2 (see [9]).**

If *A* = [*a*_*ij*_] is an *n* by *n* matrix over a commutative ring, then the standard determinant expression of *A* is

(1) $$\begin{array}{c} \det\left(A\right)=\Sigma_{\sigma \in S_{n}}\mathrm{sgn}\left(\sigma \right)a_{1\sigma \left(1\right)}a_{2\sigma \left(2\right)}\cdots a_{n\sigma \left(n\right)}, \end{array}$$

where *S*_*n*_ is the symmetric group of order *n* and sgn⁡(*σ*) = +1 if *σ* is even permutation and sgn⁡(*σ*) = −1 if *σ* is odd permutation. Here sgn⁡(*σ*)*a*_1*σ*(1)_ *a*_2*σ*(2)_ ⋯ *a*_*nσ*(*n*)_ is called a term of the determinant.

Since we do not have subtraction in a semiring, we cannot write the determinant of a matrix over a semiring in this form. We split the determinant into two parts, the positive determinant and the negative determinant.

**Definition 3 (see [9]).**

Let *A* be an *n* by *n* matrix over a commutative semiring *S*. We define the positive and the negative determinant as

(2) $$\begin{array}{c} \det_{+}\left(A\right)=\underset{\sigma \in A_{n}}{⨁}\underset{i=1}{\overset{n}{⨂}}a_{i\sigma \left(i\right)},\\ \det_{-}\left(A\right)=\underset{\sigma \in S_{n}\setminus A_{n}}{⨁}\overset{n}{\underset{i=1}{⨂}}a_{i\sigma \left(i\right)}. \end{array}$$

Here *A*_*n*_ is the alternating group of order *n*, that is, the set of all even permutations of order *n* and *S*_*n*_∖*A*_*n*_ is the set of all odd permutations of order *n*.

As such we note that the determinant of a matrix *A* over a commutative ring takes the form

(3) $$\begin{array}{c} \det\left(A\right)=\det_{+}\left(A\right)-\det_{-}\left(A\right). \end{array}$$

In the semiring case, one cannot subtract the negative determinant from the positive determinant and so the positive determinant and the negative determinant are listed as a pair. This pair is called the bideterminant.

**Definition 4 (see [10]).**

Let *A* be an *n* by *n* matrix over a commutative semiring. The bideterminant of *A* is (det⁡_+_(*A*), det⁡_−_(*A*)).

The definition of the permanent involves no subtractions; hence it carries over to the semiring case unchanged.

**Definition 5 (see [9]).**

Let *A* = [*a*_*ij*_] be an *n* by *n* matrix over a semiring; then the permanent of *A* is

(4) $$\begin{array}{c} \text{per}\left(A\right)=\underset{\sigma \in S_{n}}{⨁}a_{1\sigma \left(1\right)}a_{2\sigma \left(2\right)}\cdots a_{n\sigma \left(n\right)}. \end{array}$$

The permanent of a square matrix is the sum of its positive and negative determinants: per(*A*) = det⁡_+_(*A*) ⊕ det⁡_−_(*A*).

Finally we note that we have a canonical preorder (a reflexive transitive relation) called the difference preorder on semirings.

**Definition 6 .**

Let *S* be a semiring. We define the difference preorder ≥ on *S* as follows: if *x*, *y* ∈ *S* then *x* ≥ *y* if there exists *z* ∈ *S* such that *x* = *y* ⊕ *z*.

The difference preorder may not be a partial order. However for many semirings such as the nonnegative semiring, max-plus semiring, and any Boolean algebra or distributive lattice, the difference semiring corresponds to the natural order on the set.

## 2. The *ϵ*-Determinant and *ϵ*-Rank

In [4, 5], Tan introduced a new type of determinant for semirings. We begin with a concept formulated independently by Akian et al. in [1] and Tan in [4].

**Definition 7 (see [1, 4, 5]).**

Let *S* be a semiring. A bijection *τ* : *S* → *S* is called a symmetry if *τ*(*τ*(*a*)) = *a* for all *a* ∈ *S* and *τ*(*a* ⊗ *b*) = *a* ⊗ *τ*(*b*) = *τ*(*a*) ⊗ *b* for all *a*, *b* ∈ *S*.

The term symmetry is from [1]; this similar concept is called an *ϵ*-function in [4, 5]. We note that all of these references also required a symmetry to be additive (i.e., *τ*(*a* ⊕ *b*) = *τ*(*a*) ⊕ *τ*(*b*)). In [1], a symmetry *τ* must further satisfy *τ*(0) = 0. We have removed these conditions from the definition as we will show they follow from other properties of a symmetry. One can easily characterize all symmetries in a semiring.

**Proposition 8 .**

Let *S* be a commutative semiring. A function *τ* : *S* → *S* is a symmetry if and only if there exists an *ϵ* ∈ *S* such that *ϵ* ^2^ = 1 and *τ*(*x*) = *ϵ* ⊗ *x* for all *x* ∈ *S*.

**Proof**

Suppose *τ* : *S* → *S* is a symmetry. Let *ϵ* = *τ*(1); then *τ*(*x*) = *τ*(1 ⊗ **x**) = *ϵ* ⊗ **x**. Furthermore *ϵ* ^2^ = *ϵ* ⊗ *τ*(1) = *τ*(*ϵ* ⊗ 1) = 1. The other direction is a straightforward verification.

It follows easily from the previous proposition that if *τ* : *S* → *S* is a symmetry and *x*, *y* ∈ *S* then *τ*(*x* ⊕ *y*) = *τ*(*x*) ⊕ *τ*(*y*), *τ*(0) = 0, and *τ*(*x*) ⊗ *τ*(*y*) = *x* ⊗ *y*.

We use this observation to slightly restate the definition of an *ϵ*-determinant given in [4, 5]. We will use *ϵ* to denote the element whose square is the identity rather than a symmetry or *ϵ*-function as in [4, 5]; this allows us to use the same terminology and notation as in [4, 5] while taking advantage of the characterization of symmetries given in Proposition 8.

**Definition 9 .**

Let *S* be a commutative semiring and let *ϵ* ∈ *S* satisfy *ϵ* ^2^ = 1. Then det⁡_*ϵ*_ : *M*_*n*_(*S*) → *S* is defined as the following function: det⁡_*ϵ*_(*A*) = det⁡_+_(*A*)⊕(*ϵ* ⊗ det⁡_−_(*A*)).

We will get a distinct symmetry on *S* and hence a distinct det⁡_*ϵ*_ from every choice of *ϵ* ∈ *S* which satisfies *ϵ* ^2^ = 1. One candidate for *ϵ* that exists in every semiring is the multiplicative identity 1. If *ϵ* = 1, we get det⁡_*ϵ*_(*A*) = per(*A*).

Tan has shown that the *ϵ*-determinant satisfies two very important identities analogous to the Binet-Cauchy theorem and the Laplace expansion of the ordinary determinant. In order to state them, we introduce the following notation. Let *α* = {*α*_1_ < *α*_2_ < ⋯<*α*_*k*_} and *β* = {*β*_1_ < *β*_2_ < ⋯<*β*_*k*_} be two subsets of {1,2,…, *n*} of equal cardinality. Let *A*[*α*∣*β*] denote the *k* by *k* submatrix of *A* whose (*i*, *j*)th entry is *a*_*α*_*i*_,*β*_*j*__. We define *π*(*α*) = ∑_*j*=1_^*k*^ *α*_*j*_.

**Theorem 10 (see [5, Theorem 3.5] (the generalized Binet-Cauchy theorem)).**

Let *S* be a commutative semiring and let *ϵ* ∈ *S* satisfy *ϵ* ^2^ = 1. Let *A* ∈ *M*_*m*,*n*_(*S*), *B* ∈ *M*_*n*,*p*_(*S*), 1 ≤ *k* ≤ min⁡(*m*, *n*, *p*). Let *α* and *β* be two subsets of {1,2,…, *n*} of cardinality *k*. Then there exists a *δ* ∈ *S* such that det⁡_*ϵ*_((*AB*)[*α*, *β*]) = (⨁_*γ*⊆{1,2,…,*n*};|*γ*|=*k*_det⁡_*ϵ*_(*A*[*α*, *γ*]) ⊗ det⁡_*ϵ*_(*B*[*γ*, *β*]))⊕[*δ* ⊗ (1⊕*ϵ*)].

**Theorem 11 (see [5, Theorem 3.3] (the generalized Laplace expansion)).**

Let *S* be a commutative semiring and let *ϵ* ∈ *S* satisfy *ϵ* ^2^ = 1. If *A* ∈ *M*_*n*_(*S*) and *α*⊆{1,2,…, *n*}, then det⁡_*ϵ*_(*A*) = ⨁_*β*⊆{1,2,…,*n*};|*β*|=|*α*|_(*ϵ*)^*π*(*α*)+*π*(*β*)^ ⊗ det⁡_*ϵ*_(*A*[*α*∣*β*]) ⊗ det⁡_*ϵ*_(*A*[*α* ^*c*^∣*β* ^*c*^]).

In the case where *S* is a commutative ring and *ϵ* = −1, the *ϵ*-determinant reduces to the regular determinant and the two theorems above reduce to the usual Binet-Cauchy theorem and the Laplace expansion.

One corollary of the generalized Binet-Cauchy theorem is the following difference preorder inequality for square matrices.

**Corollary 12 .**

Let *S* be a commutative semiring and let *ϵ* ∈ *S* satisfy *ϵ* ^2^ = 1. The inequality det⁡_*ϵ*_(*AB*) ≥ det⁡_*ϵ*_(*A*) ⊗ det⁡_*ϵ*_(*B*) holds for all *A*, *B* ∈ *M*_*n*_(*S*).

The special case of this result where *S* is a Boolean algebra (and by necessity *ϵ* = 1 which means det⁡_*ϵ*_ is the permanent) has appeared in [11].

We can use Tan's generalization of the Laplace expansion to obtain a relationship between the *ϵ*-determinant of *A* + *B* and those of various submatrices of *A* and *B*.

**Corollary 13 .**

Let *n* ∈ *ℕ*, let *S* be a commutative semiring, and let *ϵ* ∈ *S* satisfy *ϵ* ^2^ = 1. The identity

(5) det⁡ϵA+B=⨁α,β⊆1,2,…,n;β=αϵπα+πβ⊗det⁡ϵ(A[α ∣ β])⊗det⁡ϵB[αc ∣ βc]

holds for all *A*, *B* ∈ *M*_*n*_(*S*).

**Proof**

Let *C*_*α*_ be the *n* by *n* matrix whose *i*th row is equal to the *i*th row of *A* if *i* ∈ *α* and whose *i*th row is equal to the *i*th row of *B* if *i* ∉ *α*. Then using the multilinearity of the *ϵ*-determinant, det⁡_*ϵ*_(*A* + *B*) = ⨁_*α*⊆{1,2,…,*n*}_det⁡_*ϵ*_(*C*_*α*_). Now we use the Laplace expansion on det⁡_*ϵ*_(*C*_*α*_), for any fixed *α*, to get

(6) det⁡ϵCα=⨁β⊆1,2,…,n;β=αϵπα+πβ⊗det⁡ϵ(A[α ∣ β])⊗det⁡ϵ(B[αc ∣ βc]).

Hence

(7) det⁡ϵA+B=⨁α,β⊆1,2,…,n;β=αϵπα+πβ⊗det⁡ϵ(A[α ∣ β])⊗det⁡ϵ(B[αc ∣ βc]).

**Definition 14 .**

Let *S* be a commutative semiring and let *A* be an *m* by *n* matrix over *S*. If 1 ≤ *k* ≤ min⁡(*m*, *n*), let *I*_*k*_^*ϵ*^(*A*) be the ideal in *S* generated by the set of all the *k* by *k* *ϵ*-minors of *A*. One defines *I*_0_^*ϵ*^(*A*) = *S* and *I*_*k*_^*ϵ*^(*A*) = {0} when *k* > min⁡(*m*, *n*).

It follows immediately from the Laplace expansion that *I*_*k*_^*ϵ*^(*A*)⊆*I*_*k*−1_^*ϵ*^(*A*) for all *k* ∈ *ℕ*.

**Definition 15 .**

Let *S* be a commutative semiring and let *ϵ* be an element of *S* such that *ϵ* ^2^ = 1 and that 1 ⊕ *ϵ* is not a unit. Let *I* be any proper ideal of *S* which contains 1 ⊕ *ϵ*. The (*ϵ*, *I*)-rank of an *m* by *n* matrix *A* (denoted by rank⁡_det⁡_^*ϵ*,*I*^(*A*)) is the largest nonnegative integer *k* such that *I*_*k*_^*ϵ*^(*A*) is not contained in *I*.

For certain semirings, it may be possible that there is no choice of *ϵ* for which 1 ⊕ *ϵ* is not a unit. An example of this is the semiring of nonnegative real numbers *ℝ* ^+^ with the usual addition and multiplication. In this case the only *ϵ* ∈ *ℝ* ^+^ satisfying *ϵ* ^2^ = 1 is 1 itself and 2 = 1 + 1 is a unit. The max-plus and max-min semirings are other examples of this. For semirings such as these, one cannot immediately define an (*ϵ*, *I*)-rank. We will examine how to handle cases like this in the last section of the paper.

The principal ideal generated by 1 ⊕ *ϵ* is a natural choice for our ideal *I*.

**Definition 16 .**

Let *S* be a commutative semiring and let *ϵ* be an element of *S* such that *ϵ* ^2^ = 1 and that 1 ⊕ *ϵ* is not a unit. Let *I*_1⊕*ϵ*_ be the principal ideal generated by 1 ⊕ *ϵ*. The *ϵ*-rank of an *m* by *n* matrix *A* (denoted by rank⁡_det⁡_^*ϵ*^(*A*)) is the largest nonnegative integer *k* such that *I*_*k*_^*ϵ*^(*A*) is not contained in *I*_1⊕*ϵ*_.

It is clear that any *I* containing 1 ⊕ *ϵ* contains *I*_1⊕*ϵ*_ and therefore rank⁡_det⁡_^*ϵ*,*I*^(*A*) ≤ rank⁡_det⁡_^*ϵ*^(*A*). Recall that the standard definition of the rank of a matrix over a ring is the size of the largest *k* for which the ideal generated by all *k* by *k* subdeterminants of the matrix is nonzero [12, page 82]. If *S* is a ring, then the (*ϵ*, *I*)-rank of a matrix *A* over *S* is equal to the standard ring-theoretic rank of the matrix *ϕ*(*A*) over the quotient ring *S*/*I* where *ϕ* is the natural entrywise quotient map.

We now examine some inequalities satisfied by these ranks that are implied by the Binet-Cauchy theorem, the Laplace expansion, and our determinant sum identity. The first is the relationship between this rank and the factor rank. We begin by reminding readers of the definition of the factor rank.

**Definition 17 .**

Let *S* be a commutative semiring and *A* ∈ *M*_*m*,*n*_(*S*). The factor rank (or Schein rank) of *A* is the smallest integer *r* for which there exists an *m* by *r* matrix *B* and an *r* by *n* matrix *C* such that *A* = *BC*. The factor rank is denoted by *f*(*A*).

**Proposition 18 .**

The inequality rank⁡_det⁡_^*ϵ*^(*A*) ≤ *f*(*A*) holds whenever *S* is a commutative semiring and *A* ∈ *M*_*m*,*n*_(*S*).

**Proof**

Let *r* = *f*(*A*), then there exist matrices *B* ∈ *M*_*m*,*r*_(*S*) and *C* ∈ *M*_*r*,*n*_(*S*) such that *A* = *BC*. Let $\hat{B}\in M_{m,r+1}(S)$ be the matrix obtained by adding a zero column to the right of *B* and let $\hat{C}\in M_{r+1,n}(S)$ be the matrix obtained by adding a zero row to the bottom of *C*. Clearly $A=\hat{B}\hat{C}$. Now we compute the *ϵ*-minors of $A(=\hat{B}\hat{C})$ of order *r* + 1, using the Binet-Cauchy theorem; that is, $\det_{\epsilon }((\hat{B}\hat{C})[\alpha ,\beta ])=\det_{\epsilon }(\hat{B}[\alpha ,\{1,2,\ldots ,r,r+1\}])⊗\det_{\epsilon }(\hat{C}[\{1,2,\ldots ,r,r+1\}$, *β*]) ⊕ *δ* ⊗ [1 ⊕ *ϵ*]. Since the first summand is 0, so *I*_*r*+1_^*ϵ*^ is contained in the ideal generated by 1 ⊕ *ϵ*. Hence rank⁡_det⁡_^*ϵ*^(*A*) ≤ *r*.

We can also prove a version of Sylvester's inequality for the (*ϵ*, *I*)-rank.

**Proposition 19 .**

Let *S* be a commutative semiring and let *ϵ* be an element of *S* such that *ϵ* ^2^ = 1 and that 1 ⊕ *ϵ* is not a unit. Let *I* be an proper ideal of *S* which contains 1 ⊕ *ϵ*. The inequality rank⁡_det⁡_^*ϵ*,*I*^(*AB*) ≤ min⁡(rank⁡_det⁡_^*ϵ*,*I*^(*A*), rank⁡_det⁡_^*ϵ*,*I*^(*B*)) holds for all *A* ∈ *M*_*m*,*n*_(*S*) and *B* ∈ *M*_*n*,*p*_(*S*).

**Proof**

Let *r* = min⁡(rank⁡_det⁡_^*ϵ*,*I*^(*A*), rank⁡_det⁡_^*ϵ*,*I*^(*B*)). If *r* ≥ min⁡(*m*, *n*, *p*) we are done so suppose *r* < min⁡(*m*, *n*, *p*) and let *α* and *β* be both arbitrary subsets of {1,2,…, *n*} of cardinality *r* + 1. Then either *I*_*r*+1_^*ϵ*^(*A*) or *I*_*r*+1_^*ϵ*^(*B*) is contained in *I*. It follows from the Binet-Cauchy theorem that det⁡_*ϵ*_(*AB*[*α*, *β*]) ∈ *I*.

It should be noted that the condition 1 ⊕ *ϵ* ∈ *I* is required for our version of Sylvester's inequality to hold; this is largely our motivation for insisting on this condition.

We also have the following rank-sum inequality.

**Proposition 20 .**

Let *S* be a commutative semiring and let *ϵ* be an element of *S* such that *ϵ* ^2^ = 1 and that 1 ⊕ *ϵ* is not a unit. Let *I* be an proper ideal of *S* which contains 1 ⊕ *ϵ*. The inequality rank⁡_det⁡_^*ϵ*,*I*^(*A* + *B*) ≤ rank⁡_det⁡_^*ϵ*,*I*^(*A*) + rank⁡_det⁡_^*ϵ*,*I*^(*B*) holds for all *A*, *B* ∈ *M*_*m*,*n*_(*S*).

**Proof**

We begin by proving the inequality in the special case where *m* = *n* and rank⁡_det⁡_^*ϵ*,*I*^(*A*) + rank⁡_det⁡_^*ϵ*,*I*^(*B*) = *n* − 1. Hence *A*, *B* ∈ *M*_*n*_(*S*). Let us suppose that *r* = rank⁡_det⁡_^*ϵ*,*I*^(*A*). This implies that *n* − *r* − 1 = rank⁡_det⁡_^*ϵ*,*I*^(*B*). We can use Corollary 13 to show that det⁡_*ϵ*_(*A* + *B*) ∈ *I*. Note that every term in the expansion of det⁡_*ϵ*_(*A* + *B*) is of a power of *ϵ* times det⁡_*ϵ*_(*A*[*α*∣*β*]) ⊗ det⁡_*ϵ*_(*B*[*α* ^*c*^∣*β* ^*c*^]) where *α* and *β* are subsets of {1,2,…, *n*} satisfying |*α* | = |*β*|. Let *k* = |*α* | = |*β*|. If *k* ≤ *r*, then *n* − *k* ≥ *n* − *r* > rank⁡_det⁡_^*ϵ*,*I*^(*B*) and since |*α* ^*c*^ | = |*β* ^*c*^ | = *n* − *k* we must have det⁡_*ϵ*_(*B*[*α* ^*c*^∣*β* ^*c*^]) ∈ *I*. Similarly, if *k* > *r*, then det⁡_*ϵ*_(*A*[*α*∣*β*]) ∈ *I*. Therefore every term in the expansion of det⁡_*ϵ*_(*A* + *B*) is in *I* and hence rank⁡_det⁡_^*ϵ*,*I*^(*A* + *B*) ≤ *n* − 1 = rank⁡_det⁡_^*ϵ*,*I*^(*A*) + rank⁡_det⁡_^*ϵ*,*I*^(*B*).

Now we prove the general case. Let *r* = rank⁡_det⁡_^*ϵ*,*I*^(*A*) + rank⁡_det⁡_^*ϵ*,*I*^(*B*). If *r* ≥ min⁡(*m*, *n*) then we are done so suppose *r* < min⁡(*m*, *n*). Now let *α* and *β* be subsets of {1,2,…, *m*} and {1,2,…, *n*}, respectively, both of cardinality *r* + 1. Then rank⁡_det⁡_^*ϵ*,*I*^((*A* + *B*)[*α*∣*β*]) ≤ rank⁡_det⁡_^*ϵ*,*I*^(*A*[*α*∣*β*]) + rank⁡_det⁡_^*ϵ*,*I*^(*B*[*α*∣*β*])≤rank⁡_det⁡_^*ϵ*,*I*^(*A*) + rank⁡_det⁡_^*ϵ*,*I*^(*B*) = *r*. Hence det⁡_*ϵ*_((*A* + *B*)[*α*∣*β*]) ∈ *I* and since (*A* + *B*)[*α*∣*β*] is an arbitrary *r* + 1 by *r* + 1 submatrix of *A* + *B*, we have rank⁡_det⁡_^*ϵ*,*I*^(*A* + *B*) ≤ *r* = rank⁡_det⁡_^*ϵ*,*I*^(*A*) + rank⁡_det⁡_^*ϵ*,*I*^(*B*).

## 3. Bijective Linear *ϵ*-Rank Preservers

In this section, we look at bijective linear operators which preserve (*ϵ*, *I*)-rank of matrices over antinegative commutative semiring.

**Definition 21 (see [2]).**

Let *S* be a commutative semiring and *A* be an *m* by *n* matrix over *S*. The term rank of *A* is the minimum number of lines (rows and columns) needed to include all nonzero entries of *A*. The term rank of a matrix *A* is denoted by *t*(*A*).

**Proposition 22 (see [2]).**

For any commutative semiring, one has *f*(*A*) ≤ *t*(*A*) whenever *A* is an *m* by *n* matrix over *S*.

Let *S* be a semiring and *A*, *B* ∈ *M*_*m*,*n*_(*S*). We write *A* ≤ *B* if there exists *C* ∈ *M*_*m*,*n*_(*S*) such that *A* ⊕ *C* = *B*. We note that the relation (≤) is a reflexive and transitive relation but not antisymmetric in general. Therefore it is a* preorder*. It is easy to check that any linear operator *T* : *M*_*m*,*n*_(*S*) → *M*_*m*,*n*_(*S*) preserves this preorder. Further, if *S* is an antinegative semiring then the term rank is a monotone function; that is, if *A* ≤ *B* then *t*(*A*) ≤ *t*(*B*).

**Definition 23 .**

Let *S* be a commutative semiring and *A*, *B* ∈ *M*_*m*,*n*_(*S*). The Schur product of *A* and *B*, denoted as *A*∘*B*, is an *m* by *n* matrix whose (*i*, *j*)th entry is *a*_*ij*_ ⊗ *b*_*ij*_.

**Definition 24 .**

Let *S* be a commutative semiring. A matrix *A* ∈ *M*_*m*,*n*_(*S*) is called a submonomial matrix if every line (row or column) of *A* contains at most one nonzero entry. A matrix *A* ∈ *M*_*n*_(*S*) is called a monomial matrix if every line (row or column) of *A* contains exactly one nonzero entry.

The concept of (*P*, *Q*, *B*) operator is a fundamental concept in the theory of linear preservers over semirings.

**Definition 25 (see [13]).**

Let *T* be a linear operator from *M*_*m*,*n*_(*S*) to itself. One says that *T* is a strong (*P*, *Q*, *B*) operator if there exist *P* ∈ *M*_*m*_(*S*), *Q* ∈ *M*_*n*_(*S*), and *B* ∈ *M*_*m*,*n*_(*S*) such that *P* and *Q* are permutation matrices, and all of the entries of *B* are units and either *T*(*X*) = *P*(*X*∘*B*)*Q* or *m* = *n* and *T*(*X*) = *P*(*X* ^*T*^∘*B*)*Q*.

We also use a theorem from the same reference. We note though there is an error earlier in [13] for the definition of the term rank, the following theorem is correct as stated as it only uses correct properties of the term rank.

**Theorem 26 (see [13, Theorem 2.12]).**

Let *S* be a commutative antinegative semiring. If rank⁡:*M*_*m*,*n*_(*S*) → *ℤ* is a function which satisfies 0 ≤ rank⁡(*A*) ≤ *t*(*A*) for all *A* ∈ *M*_*m*,*n*_(*S*) with equality whenever *A* is a submonomial matrix, then any bijective linear operator which preserves this rank function must be a strong (*P*, *Q*, *B*) operator.

Since the (*ϵ*, *I*)-rank satisfies the hypotheses of the above theorem, we now have the following corollary which classifies all bijective linear operators which preserve the (*ϵ*, *I*)-rank.

**Corollary 27 .**

Let *S* be a commutative antinegative semiring and let *ϵ* be an element of *S* such that *ϵ* ^2^ = 1 and that 1 ⊕ *ϵ* is not a unit. Let *I* be any proper ideal of *S* which contains 1 ⊕ *ϵ*. Any bijective (*ϵ*, *I*)-rank preserver on *M*_*m*,*n*_(*S*) must be a strong (*P*, *Q*, *B*) operator.

## 4. Sign Pattern Matrices and the Sign Pattern Semiring

In this section, we explore connections between the sign pattern matrices and *ϵ*-rank.

A matrix whose entries are from the set {+1, −1,0} is called a* sign pattern matrix*. If *A* = [*a*_*ij*_] is a real matrix, then the sign pattern of *A* is obtained from *A*, by replacing each entry by its signs [14, 15]. The sign pattern of *A* is denoted by Sg(*A*) = [sg(*a*_*ij*_)], where

(8) $$\begin{array}{c} \text{sg}\left(a_{ij}\right)=\left\{\begin{array}{cc} 0 & \text{if}\;a_{ij}=0\\ +1 & \text{if}\;a_{ij}>0\\ -1 & \text{if}\;a_{ij}<0. \end{array}\right. \end{array}$$

Thus in a sign pattern matrix all we know is the sign of each entry. We do not know the exact values of the entries. We denote the set of all *n* by *n* sign pattern matrices by *Q*_*n*_. Sometimes we may not know the signs of certain entries, so a new symbol, #, has been introduced to denote such entries.

**Definition 28 (see [16]).**

The generalized sign pattern matrices are the matrices over the set {+1, −1,0, #}, where # corresponds to entries where the sign is unknown.

The set {+1, −1,0, #} can be viewed as a semiring. If *S* = {+1, −1,0, #}, then (*S*, ⊕, ⊗) is a commutative semiring with identity, where the operations of addition and multiplication are defined as follows:

(9) $$\begin{array}{c} \begin{array}{ccccc} ⊕ & 0 & +1 & -1 & \#\\ 0 & 0 & +1 & -1 & \#\\ +1 & +1 & +1 & \# & \#\\ -1 & -1 & \# & -1 & \#\\ \text{\#} & \# & \# & \# & \# \end{array}\\ \begin{array}{ccccc} ⊗ & 0 & +1 & -1 & \#\\ 0 & 0 & 0 & 0 & 0\\ +1 & 0 & +1 & -1 & \#\\ -1 & 0 & -1 & +1 & \#\\ \text{\#} & 0 & \# & \# & \# \end{array} \end{array}$$

Clearly all the properties of a semiring are satisfied where 0 is the additive identity and +1 is multiplicative identity. Here +1 and −1 are the units of *S*. More about the sign pattern semiring can be found in [17].

**Definition 29 (see [14]).**

Let *A* be a real matrix. The qualitative class of *A* is *Q*(*A*), the set of all real matrices with the same sign pattern as *A*.

**Definition 30 (see [14]).**

A sign pattern matrix *A* is called sign-nonsingular (SNS) if every matrix in its qualitative class is nonsingular.

For matrices over the sign pattern semiring, we can give a more concrete interpretation of the *ϵ*-rank. The sign pattern semiring has only two elements whose square is the identity, namely, 1 and −1. The ideal generated by 1 = 1 + 1 is the entire semiring but # = 1 + −1 generates the unique proper ideal {#, 0}. Therefore −1 is the only available choice for *ϵ* and we have a unique *ϵ*-rank. Hence det⁡_*ϵ*_(*A*) = det⁡_+_(*A*)⊕(−1 ⊗ det⁡_−_(*A*)). It is easy to show that an *n* by *n* sign pattern matrix has *ϵ*-rank *n* if and only if it is an SNS matrix. Hence the *ϵ*-rank of a sign pattern matrix *A* is the largest integer *k* for which there exists a *k* by *k* SNS submatrix of *A*.

## 5. Sublocal Semirings

It was remarked earlier that rank⁡_det⁡_^*ϵ*,*I*^(*A*) ≤ rank⁡_det⁡_^*ϵ*^(*A*). In other words amongst the family of (*ϵ*, *I*)-ranks, choosing *I* to be the ideal generated by 1 ⊕ *ϵ* gives us the largest possible rank function from this family. The minimal rank functions from this family arise from the choice of *I* to be a maximal ideal which contains 1 ⊕ *ϵ*. In general, there may be many maximal ideals. In this section, we will look at semirings which have a unique maximal ideal. We will use the term sublocal semiring to denote a semiring which has a unique maximal ideal. Sublocality in semirings is essentially the straightforward generalization of the very useful concept of locality in rings. We use the term sublocal semiring because the term local semiring has been used to define a slightly different concept.

**Definition 31 (see [18]).**

An ideal *I* of a commutative semiring *S* is called a *k*-ideal if for any *y* ∈ *S* with *x*, *x* + *y* ∈ *I* one has *y* ∈ *I*.

We note that if we consider a commutative ring *R* to be semiring, the semiring ideals of *R* are exactly the semiring *k*-ideals of *R* which are also exactly the ring ideals of *R*.

**Definition 32 (see [18]).**

A proper ideal *I* of a commutative semiring *S* is called a maximal (resp., *k*-maximal) ideal if there exists no other proper ideal (resp., *k*-ideal)  *J* such that *I* ⊂ *J*.

**Definition 33 (see [18]).**

Let *S* be a commutative semiring. One says that *S* is a local semiring if *S* has only one *k*-maximal ideal.

Now we will define sublocal semirings using maximal ideals instead of *k*-maximal ideals. This is useful as some semirings do not have proper *k*-ideals. For example, the sign pattern semiring has only one proper ideal {0, #} and this is not a *k*-ideal.

**Definition 34 .**

Let *S* be a commutative semiring. One says that *S* is a sublocal semiring if *S* has only one maximal ideal.

We note that both local and sublocal semirings are direct but different semiring generalizations of the concept of a local ring. Local semirings have been useful in semiring theory; see [18] for examples of this. We will show that sublocality is a useful property as well. There are many examples of sublocal semirings; we list some of the notable ones. The sign pattern semiring is a sublocal semiring having only one maximal ideal *I* = {0, #}. We note that this maximal ideal is contained in the proper subsemiring *P* = {0, +1, #}; *P* however fails to be an ideal in the sign pattern semiring. The set of all natural numbers, *ℕ* = {0,1, 2,…}, forms a sublocal semiring whose only one maximal ideal *I* = {*ℕ*/{1}}. All chain semirings are sublocal semirings with *S*/{1} as a unique maximal ideal. A semifield is a commutative semiring in which all elements except 0 have a multiplicative inverse. (The Boolean and max-plus semirings are examples of semifields.) All semifields are sublocal semirings as the zero ideal is the unique maximal ideal.

We begin with the following elementary lemma whose proof is identical to the corresponding result for rings.

**Lemma 35 (see [18]).**

An element *a* of a commutative semiring *S* is a unit of *S* if and only if *a* lies outside all maximal ideals of *S*.

**Proof**

Let *a* be a unit of *S*; then the ideal generated by *a* must be *S* itself and hence *a* lies outside all maximal ideals of *S*. If *a* is not a unit of *S*, then 1 ∉ *aS* and hence there exits a maximal ideal *M* of *S* such that *a* ∈ *aS*⊆*M*.

One of the key results of [18] is that a semiring is local if the set of all of its nonunits forms a *k*-ideal. The analog for sublocal semirings is an easy consequence of Lemma 35.

**Corollary 36 .**

A commutative semiring *S* is a sublocal semiring if and only if the set of all nonunits of *S* forms an ideal.

Since every *k*-ideal is an ideal, it follows that every local semiring is a sublocal semiring. The converse is false. Consider the nonnegative integers *ℕ* with the usual addition and multiplication. The unique maximal ideal is *ℕ*∖{1}; the maximal *k*-ideals are of the form *pℕ* for any prime *p*.

Since the set of nonunits in any sublocal semiring is an ideal, the nonunits are closed under addition. Hence we have the following observation which will prove useful later on.

**Corollary 37 .**

Let *S* be a sublocal semiring. Let *a*_1_, *a*_2_ ∈ *S*. If *a*_1_ ⊕ *a*_2_ is a unit of *S*, then either *a*_1_ or *a*_2_ is a unit of *S*.

## 6. Symmetrized Semirings

The ranks introduced in the previous sections all require an element *ϵ* satisfying the condition that *ϵ* ^2^ = 1 and 1 ⊕ *ϵ* is not a unit. Such an element may not exist in a given semiring; the max-min and max-plus semirings are examples of semiring which lack an *ϵ*. Fortunately, there is a known construction which allows us to append such an element. This construction is from [8], in which it was applied to the max-plus semiring. In this paper we explore applications of this construction both to general semirings and to the specific examples such as the Boolean semiring and the sign pattern semiring.

If (*S*, ⊕, ⊗) is a commutative semiring then *S* ^2^ = {(*a*, *b*)∣*a*, *b* ∈ *S*} is also a commutative semiring with addition and multiplication defined as follows: for all *a*, *b*, *c* and *d* ∈ *S*,

(10) $$\begin{array}{c} \left(a,b\right)⊕\left(c,d\right)=\left(a⊕c,b⊕d\right),\\ \left(a,b\right)⊗\left(c,d\right)=\left(\left(a⊗c\right)⊕\left(b⊗d\right),\left(b⊗c\right)⊕\left(a⊗d\right)\right). \end{array}$$

We can see that all the properties of a semiring are satisfied with (0, 0) being the additive identity of *S* ^2^. Essentially this construction allows us to append an element *ϵ* = (0, 1) with the property *ϵ* ^2^ = 1 to the semiring *S* in a natural way giving us a way to apply the *ϵ*-determinant theory to semirings which do not have nontrivial self inversive elements. The ideal in *S* ^2^ generated by (1, 1) = (1, 0) + *ϵ* is Δ = {(*x*, *x*) : *x* ∈ S} which we will call the diagonal ideal. The *ϵ*-determinant in this case is the standard bideterminant and the *ϵ*-rank is the standard determinantal rank defined as follows.

**Definition 38 .**

Let *A* be an *m* by *n* matrix over a commutative semiring *S*. The determinantal rank of *A* is the largest *k* for which there exists *B*, a *k* by *k* submatrix of *A* with det⁡_+_(*B*) ≠ det⁡_−_(*B*).

The determinantal rank has been much studied (see [1], for instance). Our results in Section two generalize the known results on the determinantal rank of max-plus matrices to the (*ϵ*, *I*)-rank of matrices over general semirings.

**Remark 39 .**

Recall that *β* denotes Boolean semiring which has two elements {0,1}. Then *β* ^2^ = {(0,0), (1,0), (0,1), (1,1)} is also a semiring with the addition and the multiplication defined above for *S* ^2^. Moreover it is isomorphic to the sign pattern semiring as (0,0) = 0, (1,0) = +1, (0,1) = −1, and (1,1) = #.

We complete our paper by showing that the symmetrized semiring *S* ^2^ inherits some important properties from *S*.

**Theorem 40 .**

Let *S* be a commutative semiring. If *S* is antinegative and has no zero divisors then *S* ^2^ is also antinegative and has no zero divisors.

**Proof**

Since *S* is antinegative, only 0 has an additive inverse. Let us suppose that (*a*_1_, *b*_1_) ∈ *S* ^2^ has an additive inverse, so there exists (*a*_2_, *b*_2_) ∈ *S* ^2^, such that (*a*_1_, *b*_1_)⊕(*a*_2_, *b*_2_) = (0, 0). Consequently *a*_1_ ⊕ *a*_2_ = 0 and *b*_1_ ⊕ *b*_2_ = 0. Since *S* is antinegative so *a*_1_ = *a*_2_ = *b*_1_ = *b*_2_ = 0. Hence (*a*_1_, *b*_1_) = (0, 0). Thus only the additive identity has an additive inverse in *S* ^2^ which means *S* ^2^ is an antinegative semiring. Now suppose that (*a*_1_, *b*_1_) ∈ *S* ^2^ is a zero divisor, so there exists a nonzero (*a*_2_, *b*_2_) ∈ *S* ^2^, such that (*a*_1_, *b*_1_)⊗(*a*_2_, *b*_2_) = (0, 0). It follows that ((*a*_1_ ⊗ *a*_2_)⊕(*b*_1_ ⊗ *b*_2_)) = 0 and ((*a*_1_ ⊗ *b*_2_)⊕(*a*_2_ ⊗ *b*_1_)) = 0. Since *S* is antinegative, *a*_1_ ⊗ *a*_2_ = 0 and *b*_1_ ⊗ *b*_2_ = 0. Also *S* has no zero divisors so either *a*_1_ = 0 or *a*_2_ = 0 and either *b*_1_ = 0 or *b*_2_ = 0; combining this with ((*a*_1_ ⊗ *b*_2_)⊕(*a*_2_ ⊗ *b*_1_)) = 0, we get either (*a*_1_, *b*_1_) = (0, 0) or (*a*_2_, *b*_2_) = (0, 0). Hence *S* ^2^ has no zero divisors.

If *S* is a semiring, we let *U*(*S*) denote the set of units of *S*. There is a very close relation between the units of *S* and the units of *S* ^2^.

**Lemma 41 .**

If S is a commutative antinegative semiring with no zero divisors then *U*(*S* ^2^) = {(*x*, 0) : *x* ∈ *U*(*S*)}⋃{(0, *x*) : *x* ∈ *U*(*S*)}.

**Proof**

Let *x* be a unit in *S*. Then there exists a nonzero element *a* in *S* such that *x* ⊗ *a* = 1. Consider (*x*, 0)⊗(*a*, 0) = (*x* ⊗ *a*, 0) = (1, 0) and (0, *x*)⊗(0, *a*) = (*x* ⊗ *a*, 0) = (1, 0). Consequently (*x*, 0) and (0, *x*) are units of *S* ^2^. Now we have to prove that these are the only units for *S* ^2^. Suppose (*x*, *y*) ∈ *S* ^2^ is a unit in *S* ^2^. Then there exists a nonzero element (*a*, *b*) ∈ *S* ^2^ such that (*x*, *y*)⊗(*a*, *b*) = (1, 0). Thus ((*a* ⊗ *x*)⊕(*b* ⊗ *y*), (*b* ⊗ *x*)⊕(*a* ⊗ *y*)) = (1, 0). Consequently (*a* ⊗ *x*)⊕(*b* ⊗ *y*) = 1 and (*b* ⊗ *x*)⊕(*a* ⊗ *y*) = 0. Since (*b* ⊗ *x*)⊕(*a* ⊗ *y*) = 0 and *S* is an antinegative semiring, so *b* ⊗ *x* = 0 and *a* ⊗ *y* = 0. Also given that *S* has no zero divisors it follows that either *b* = 0 or *x* = 0 (note that both *b* and *x* cannot be zero because (*a* ⊗ *x*)⊕(*b* ⊗ *y*) = 1) and either *a* = 0 or *y* = 0 (here also both *a* and *y* cannot be zero because (*a* ⊗ *x*)⊕(*b* ⊗ *y*) = 1). Since (*x*, *y*) and (*a*, *b*) are nonzero elements of *S* ^2^ so the units of *S* ^2^, (*x*, *y*), have only two choices which are (*x*, 0) and (0, *y*). Putting (*x*, *y*) = (*x*, 0) in (*a* ⊗ *x*)⊕(*b* ⊗ *y*) = 1, we get *a* ⊗ *x* = 1, and this means that *x* is a unit of *S*. Putting (*x*, *y*) = (0, *y*) in (*a* ⊗ *x*)⊕(*b* ⊗ *y*) = 1, we get *b* ⊗ *y* = 1, and this means that *y* is a unit of *S*. Thus all the units in *S* ^2^ are of the type (*x*, 0) and (0, *x*) where *x* is a unit in *S*.

We can now prove that if *S* is a sublocal antinegative semiring with no zero divisors then so is *S* ^2^.

**Theorem 42 .**

If *S* is a sublocal antinegative semiring with no zero divisors then *S* ^2^ is also a sublocal antinegative semiring with no zero divisors.

**Proof**

Suppose *S* is a sublocal antinegative semiring with no zero divisors; then by Theorem 40, *S* ^2^ is an antinegative semiring with no zero divisors. Hence we only need to prove that *S* ^2^ is sublocal; we show this by proving that the set of all nonunits in *S* ^2^ forms an ideal of *S* ^2^. Let *M* = {(*a*, *b*) : where  (*a*, *b*)  is  not  a  unit  of  *S* ^2^} be the set of all nonunits of *S* ^2^. Let (*a*_1_, *b*_1_) and (*a*_2_, *b*_2_) ∈ *M* such that (*a*_1_, *b*_1_)⊕(*a*_2_, *b*_2_) = (*x*, 0), where *x* is a unit in *S*. Hence *a*_1_ ⊕ *a*_2_ = *x* and *b*_1_ ⊕ *b*_2_ = 0. Since *S* is antinegative so *b*_1_ = 0 and *b*_2_ = 0, and also *a*_1_ ⊕ *a*_2_ = *x*, where *x* is a unit in *S* so (using Corollary 37) either *a*_1_ is a unit or *a*_2_ is a unit. Thus either (*a*_1_, *b*_1_) = (*a*_1_, 0), where *a*_1_ is a unit in *S*, or (*a*_2_, *b*_2_) = (*a*_2_, 0), where *a*_2_ is a unit in *S*. It follows that either (*a*_1_, *b*_1_) is a unit or (*a*_2_, *b*_2_) is a unit in *S* ^2^, which is a contradiction to the fact that both (*a*_1_, *b*_1_) and (*a*_2_, *b*_2_) ∈ *M*. Thus the sum of nonunits in *S* ^2^ is a nonunit. A similar argument works if (*a*_1_, *b*_1_)⊕(*a*_2_, *b*_2_) = (0, *x*), where *x* is a unit in *S*. Now suppose that for (*a*, *b*) ∈ *M* and (*s*_1_, *s*_2_) ∈ *S* ^2^ we have (*a*, *b*)⊗(*s*_1_, *s*_2_) = (*x*, 0), where *x* is a unit in *S*. Then (*a* ⊗ *s*_1_)⊕(*b* ⊗ *s*_2_) = *x* and (*a* ⊗ *s*_2_)⊕(*b* ⊗ *s*_1_) = 0. Since *S* is antinegative so *a* ⊗ *s*_2_ = 0 and *b* ⊗ *s*_1_ = 0, and also *S* has no zero divisors so either *a* = 0 or *s*_2_ = 0 and either *b* = 0 or *s*_1_ = 0. Clearly (*a*, *b*) or (*s*_1_, *s*_2_) cannot be (0, 0) since (*a* ⊗ *s*_1_)⊕(*b* ⊗ *s*_2_) = *x*, so we have (*a*, *b*) = (0, *b*) or (*a*, 0). Further *x* is a unit in *S* and (*a* ⊗ *s*_1_)⊕(*b* ⊗ *s*_2_) = *x*, so either *a* ⊗ *s*_1_ is a unit in *S* or *b* ⊗ *s*_2_ is a unit in *S*. Hence either *a* is a unit in *S* or *b* is a unit in *S*. We get (*a*, *b*) = (0, *b*) or (*a*, 0) is a unit in *S* ^2^, which is a contradiction to the fact that (*a*, *b*) ∈ *M*. Thus (*a*, *b*)⊗(*s*_1_, *s*_2_) ∈ *M* for all (*a*, *b*) ∈ *M* and (*s*_1_, *s*_2_) ∈ *S* ^2^. Hence the set of all nonunits in *S* ^2^ forms an ideal of *S* ^2^. Consequently *S* ^2^ is a sublocal semiring. A similar argument works if (*a*, *b*)⊗(*s*_1_, *s*_2_) = (0, *x*), where *x* is a unit in *S*.
